# Efficient Biosynthesis of Gastrodin by UDP-Glycosyltransferase from *Rauvolfia serpentina*

**DOI:** 10.4014/jmb.2501.01002

**Published:** 2025-03-26

**Authors:** Lin Ge, Yu Xia, Wenxin Xu, Ruobing Jia, Tingting Zhang

**Affiliations:** 1 College of Biopharmacy, Suzhou Chien-Shiung Institute of Technology, Taicang 215411, P.R. China; 2 Jiangsu Provincial Novel Anti-tumor Targeted Drug Conjugate Engineering Research Center, Suzhou 215411, P.R. China

**Keywords:** Gastrodin, UDP-glycosyltransferase, *Rauvolfia serpentina*, biosynthesis

## Abstract

Gastrodin, the primary bioactive constituent of *Gastrodia elata*, possesses numerous remarkable pharmacological properties. In this investigation, UDP-glycosyltransferase from *Rauvolfia serpentina* (RsUGT) was expressed, subsequently purified and characterized. The maximum yield of the enzyme was 17.57 mU/ml and possessed a relative molecular weight of approximately 77.7 kDa. Utilizing GST affinity resin, RsUGT was purified 20.8-fold, with an overall recovery rate of 58.6% and specific activity of 79.2 mU/mg. The optimal temperature and pH for RsUGT was identified as 40°C and 10.0, respectively. Notably, 2% DMSO could increase the RsUGT activity by 12.15%. The Michaelis-Menten constants *K*_M_ and *V*_max_ were determined to be 0.50mM and 171.60 mU/mg. By optimizing the conditions for the enzymatic biosynthesis of gastrodin by RsUGT, the highest gastrodin production was 285.35 mg/l, accompanied by a molar conversion rate of 99.67%. In addition, the conditions of gastrodin biosynthesis by recombinant strain BL-RsUGT were also studied. The highest gastrodin production was 225.99 mg/l, and the corresponding *p*HBA conversion rate was 98.00%. These findings confirmed the promising potential of RsUGT in the production of gastrodin.

## Introduction

Gastrodin, a phenolic glycoside chemically identified as 4-hydroxybenzyl alcohol-4-O-β-D-glucopyranoside, stands as the primary bioactive constituent of the esteemed herb *Gastrodia elata*. This compound exhibits favorable clinical outcomes in addressing cardiovascular diseases [1] and is extensively utilized as an adjunctive therapy for vertigo, neuralgia, headache, neurasthenia, and epilepsy, demonstrating notable therapeutic efficacy without evident toxicity or side effects [2-5]. Furthermore, gastrodin possesses a series of pharmacological activities, including anti-inflammatory [6], anti-anxiety [7], anti-alcoholic liver injury [8], antioxidant [9], anti-obesity properties, neuroprotection, and memory enhancement [10]. Currently, the market boasts 44 types of drugs and health products that feature gastrodin as their core component [11]. Traditionally, gastrodin production has relied on plant extraction and compound synthesis methods [12, 13]. However, the scarcity of wild *G. elata* resources and the intricacies of artificial cultivation pose significant challenges [14]. Additionally, the low concentration of gastrodin in *G. elata* plants (mass fraction < 0.7%) [11] elevates extraction costs and prolongs the extraction process. The complex structure of gastrodin further complicates chemical synthesis, often yielding numerous analogs that are difficult to separate. The use of toxic phenols, phosphates, and bromides in the synthesis process also contributes to severe environmental pollution [15]. In contrast, biological methods offer distinct advantages, including high specificity, mild reaction conditions, and minimal pollution. Consequently, recent years have witnessed a surge in reports on the biological preparation of gastrodin [11, 13, 15-17], making it a focal point of research.

Glycosyltransferases are crucial carbohydrate active enzyme, which can catalyze the formation of glycosidic bonds between specific small molecules and uridine 5'-diphosphate (UDP) sugar [18]. Although the natural UDP-glycosyltransferase of gastrodin biosynthesis was still unclear, it had been reported that some UDP-glycosyltransferases can convert *p*-hydroxybenzyl alcohol (*p*HBA) into gastrodin. For example, UGT73B6 from *Rhodiola sachalinensis* can convert *p*HBA (2 mM) into gastrodin [13], but the conversion rate was only 9%. Cui *et al*. found that the combination of itUGT2 from *Indigofera tinctoria* and GmSuSy from *Glycine max* can convert *p*HBA (2 mM) into gastrodin [16], with a conversion rate of 93% and a gastrodin yield of 535 mg/l. Xia *et al*. found that AtUGT from *Arabidopsis thaliana* can convert (10 mM) *p*HBA into gastrodin [17], with a conversion rate of 94.34% and a gastrodin yield of 2.67 g/l. Guo *et al*. found that RrUGT3 from *Rhodiola rosea* can convert 0.5 mM *p*HBA into gastrodin [19], with a conversion rate of 99.1% and a gastrodin yield of 142 mg/l. SlyUGT from *Solanum lycopersicum* can convert *p*HBA (2 mM) into gastrodin [20], with a conversion rate of 97.82% and a gastrodin yield of 559.83 mg/l. The amino acid sequence of UDP-glycosyltransferase gene from *Rauvolfia serpentina* (RsUGT) and SlyUGT gene from *Solanum lycopersicum* have 68.95% homology. RsUGT may have special enzymatic properties. Therefore, in this study, the RsUGT was efficiently expressed in *Escherichia coli* BL21(DE3), and the expression conditions were optimized. The enzymatic properties of RsUGT were characterized, and the conditions for the enzymatic biosynthesis of gastrodin by RsUGT were investigated. Furthermore, the conditions of gastrodin biosynthesis by recombinant strain BL-RsUGT were also explored. The results indicated that RsUGT possessed significant potential for application in the biosynthesis of gastrodin.

## Materials and Methods

### Materials

Competent cells of the *E. coli* strain BL21(DE3) were procured from Beijing TransGen Biotech Co. Ltd., (China) Plasmid pGEX-2T was sourced from GE Healthcare. The modified Bradford protein assay kit and dimethyl sulfoxide (DMSO) were obtained from Shanghai Beyotime Biotech Co. Ltd., (China) Gastrodin, *p*-hydroxybenzyl alcohol (*p*HBA), UDP-glucose, and isopropyl-β-D-thiogalactopyranoside (IPTG) were obtained from Shanghai Aladdin Biotech Co. Ltd., (China) The GSTSep Glutathione Agarose Resin GST, gravity chromatography columns, and reduced glutathione (GSH) were obtained from Shanghai Yeasen Biotech Co. Ltd., (China) The Protein Marker, FlyCut *BamH* I and FlyCut *EcoR* I were obtained from Beijing TransGen Biotech Co. Ltd.,(China).

### Plasmid Constructions

The gene encoding RsUGT from *Rauvolfia serpentina* (GenBank No. Q9AR73.1) was synthesized to optimize for *E. coli* codon usage at Suzhou GENEWIZ Biotech Co. Ltd., (China). The synthesized gene RsUGT was then inserted into the vector pGEX-2T using FlyCut *BamH* I and *EcoR* I, resulting in the creation of the recombinant vector pGEX-RsUGT.

### Optimizing Expression Conditions of Recombinant Enzyme RsUGT

Recombinant vector pGEX-RsUGT was employed to transform *E. coli* BL21(DE3) competent cells, resulting in the recombinant strain BL-RsUGT. The recombinant strains were cultured in 6 ml of LB medium supplemented with 100 mg/l ampicillin and incubated overnight at 37°C. This culture was used as the seed liquid, which was subsequently inoculated into 50 ml of fresh LB medium containing 100 mg/l ampicillin at an inoculation rate of 2%. When the OD600 reached 1.0, 0.1 mM IPTG was added, and the culture was induced at 37°C. To optimize enzyme production conditions, the effects of various expression conditions on enzyme yield were investigated, including different induction temperatures (20, 25, 30, and 37°C), IPTG concentrations (0, 0.05, 0.1, 0.2, 0.3, 0.4, and 0.5 mM), OD600 values at induction (0.8, 1.0, 1.2, 1.5, 1.8, and 2.0), and induction durations (12, 18, 24, 36, 42, and 48 h).

### Purification and Concentration Determination of Recombinant Enzyme RsUGT

The recombinant *E. coli* cells, which were cultured under the optimal expression and induction conditions, were collected by centrifugation at 4°C and 10,000 g for 5 min. The cells were then clarified, resuspended in PBS buffer (pH 7.4), and subjected to lysis using an ultrasonic crusher. The supernatants obtained were loaded onto a GST affinity column containing GSTSep Glutathione Agarose Resin at a flow rate of 2 ml/min. Subsequently, the PBS buffer (pH 7.4) was used for impurity protein washing at a flow rate of 2 ml/min. Finally, the eluted protein was carried out with PBS buffer (pH 7.4) containing 10 mM glutathione (GSH) at a flow rate of 2 ml/min. The eluted protein was dialyzed with PBS buffer (pH 7.4) at 4°C for 4 times, 2 h each time. Subsequently, glycerol with a final concentration of 30% was added to the enzyme solution, and then it was stored at -80°C. The resulting proteins were examined by SDS-PAGE and analyzed by a gel imager.

Protein concentrations were assayed according to Bradford method by using a Bradford protein assay kit with bovine serum albumin as the standard protein. The protein sample were reacted with Bradford reagent at 30°C for 10 min, and then the reaction sample were detected at 595 nm [21].

### Activity Assay of Recombinant Enzyme RsUGT

RsUGT activity was evaluated using *p*HBA as a substrate in a reaction mixture with a total volume of 100 μl. The reaction components included 1 mM *p*HBA, 50 mM glycine buffer (pH 10.0), 1 mM UDP-glucose, and an appropriate amount of purified RsUGT. The samples were carried out at 40°C for 1 h and were subsequently terminated by adding 4 μl of 10% trifluoroacetate in water and 900 μl of methanol. The reaction products were then analyzed using high-performance liquid chromatography (HPLC). One unit of enzyme activity was defined as the amount of enzyme necessary to biosynthesize 1 μmol of gastrodin of per h under the assay conditions.

### Characterization of Recombinant Enzyme RsUGT

The optimum pH for RsUGT was determined using 50 mM PB buffer (6.0, 6.5, 7.0, 7.5, and 8.0), Tris-HCl buffer (7.0, 7.5, 8.0, 8.5, 9.0, and 9.5), and glycine buffer (8.5, 9.0, 9.5, 10.0, and 10.5) at 40°C for 1 h. The pH resulting in the highest enzyme activity was considered the optimal pH. Similarly, the optimal temperature was determined using glycine buffer at various temperatures (25, 30, 35, 40, 45, and 50°C) for 1 h at the optimal pH, with the temperature yielding the highest activity being the optimal temperature.

The thermal stability of RsUGT was determined by measuring the inactivation rate of RsUGT enzyme at 35°C, 40°C, and 45°C when the pH value of the glycine buffer was 10.0. The pH stability of RsUGT was determined by mixing the enzyme solution and buffer solution with the above three different pH buffers in a ratio of 1:1, then keeping the temperature at 35°C for 3 h, and finally determining the residual enzyme activity of RsUGT.

Common metal ions and chemical reagents were selected to assess their impact on enzyme activity, with final concentrations in the reaction system maintained at 1 mM. The activity was determined as described above and was expressed as a percentage of the activity observed in the absence of the chemical agents and metal cations.

To investigate the effect of dimethyl sulfoxide (DMSO) on RsUGT activity, various concentrations of DMSO (ranging from 1% to 12%) were added to the reaction mixture. The activity was determined as described above and was expressed as a percentage of the activity observed in the absence of DMSO.

The kinetic parameters of RsUGT, namely *K*_M_ and *V*_max_, were determined from Michaelis-Menten plots by assessing the initial reaction rates using various concentrations of *p*HBA (0.2, 0.4, 0.5, 1, 2, 3, and 4 mM) at pH 10.0 and 40°C for 1 h.

### Optimizing the Conditions for the Enzymatic Biosynthesis of Gastrodin

The reaction system, comprising 100 μl of a mixture containing 1 mM *p*HBA, 1 mM UDP-glucose, 2 mU/ml RsUGT, and 50 mM glycine buffer (pH 10.0), was conducted in a metal bath to optimize the biosynthesis conditions of gastrodin. Various parameters were tested, including different pH values of glycine buffer (ranging from 8.5 to 10.5), temperatures (ranging from 25 to 50°C), UDP-glucose concentrations (ranging from 1 to 6 mM), RsUGT concentrations (ranging from 1 to 7 mU/ml), and reaction times (ranging from 0 to 7 h). The reaction was initiated by the addition of purified RsUGT. To terminate the reaction, 4 μl of 10% trifluoroacetate water and 900 μl of methanol were added to the samples.

### Optimization of the Conditions of Gastrodin Production by BL-RsUGT

Recombinant strains were inoculated into 6 ml of fresh TB medium containing 100 mg/l ampicillin and were grown at 37°C until the absorbance at 600 nm reached 1.0. *p*HBA was dissolved at a concentration of 100 g/l in dimethyl sulfoxide (DMSO) as a stock solution. *p*HBA and IPTG were added to final concentrations of 0.2 g/l and 0.1 mM, respectively. The fermentation broths were incubated at 30°C and 180 rpm for 18 h. 200 μl of culture broth was taken out, and 4 μl of 10% trifluoroacetic acid water and 800 μl of methanol were added to it. The supernatant was harvested by centrifugation at 12,000 ×*g* for 10 min and analyzed by high performance liquid chromatography (HPLC).

In order to obtain the optimal conditions for gastrodin production, the effects of different culture conditions on gastrodin production were studied, including different *p*HBA addition time (1, 2, 3, 5, and 7 h), induction temperatures (16, 20, 25, 30, and 37°C), IPTG concentrations (0.00, 0.02, 0.04, 0.06, 0.08, 0.10, and 0.12 mM), *p*HBA concentrations (0.08, 0.10, 0.12, 0.14, 0.16, 0.18, and 0.20 g/l), and induction durations (6, 12, 18, 24, 30, 36, and 42 h).

### HPLC Analysis

HPLC analysis of *p*HBA and gastrodin was conducted using an Agilent HPLC 1200 system equipped with an Agela Innoval C18 column (4.6 × 250 mm, i.d., 5 μm). The mobile phases utilized in the HPLC were methanol (mobile phase A) and 0.1% trifluoroacetic acid water (mobile phase B). The chromatographic conditions were set as follows: from 0 to 6 min., the composition was 10% solvent A and 90% solvent B; from 6 to 21 min., it was adjusted to 90% solvent A and 10% solvent B; it remained constant until 25 min.; and then, from 25 to 35 min., it returned to 10% solvent A and 90% solvent B. The flow rate was maintained at 1.0 ml/min., the column temperature was set at 30°C, and detection was achieved by monitoring the absorbance at a wavelength of 225 nm.

### Statistical Analysis

The data were presented as means ± standard deviation (SD) and analyzed using Student's *t*-test to identify any statistically significant differences. All statistical analyses were performed using SPSS version 10.0 software. Differences were considered statistically significant when the *P* value was less than 0.01.

## Results and Discussion

### Optimization of Culture Conditions

**Fig. 1:**
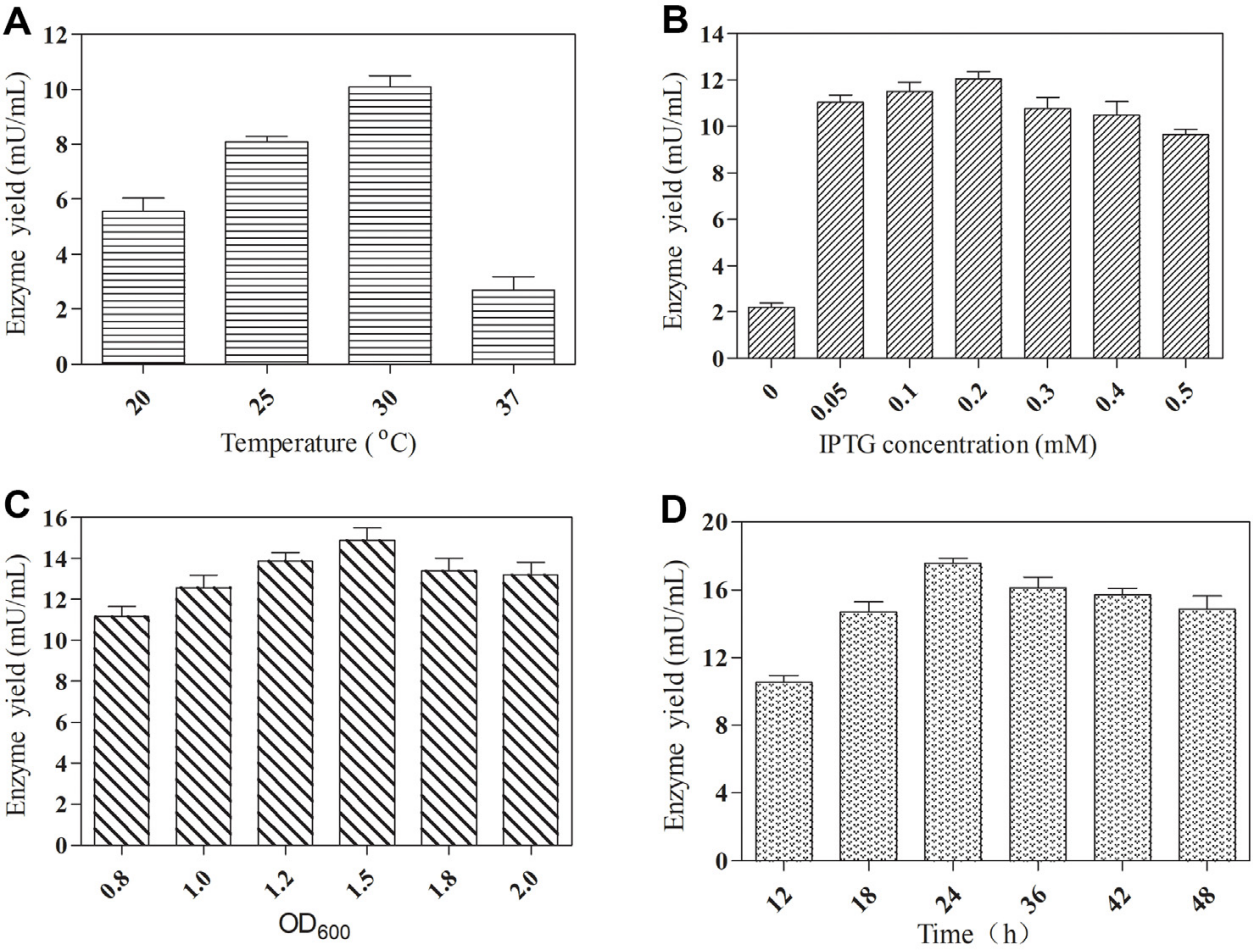
Optimization of culture conditions of RsUGT: (A) induction temperature; (B) IPTG concentration; (C) OD600; (D) induction time.

The expression of plant-derived genes in *E. coli* often results in the formation of inclusion bodies. To investigate the impact of induction temperature on RsUGT production, various temperatures were studied. As illustrated in Fig. 1A, the optimal induction temperature was determined to be 30°C. The concentration of the inducer IPTG also significantly influences recombinant protein production. Therefore, the effects of various IPTG concentrations on the production of RsUGT by recombinant strain BL-RsUGT were studied at 30°C. The results showed that the optimum IPTG concentration was 0.2 mM (Fig. 1B), resulting in a 4.5-fold increase in RsUGT yield compared to the non-induced control. Additionally, the optimal OD_600_ value was determined to be 1.5 (Fig. 1C), which indicated that the maximum balance between bacterial growth and protein expression can be achieved under this condition. Furthermore, the maximum enzyme activity was observed 24 h post-induction (Fig. 1D), potentially due to protease degradation of the enzyme after this time point. Under the optimum culture conditions, the maximum enzyme activity of the target protein RsUGT was 17.57 mU/ml.

### Purification and Characterization of RsUGT

**Fig. 2:**
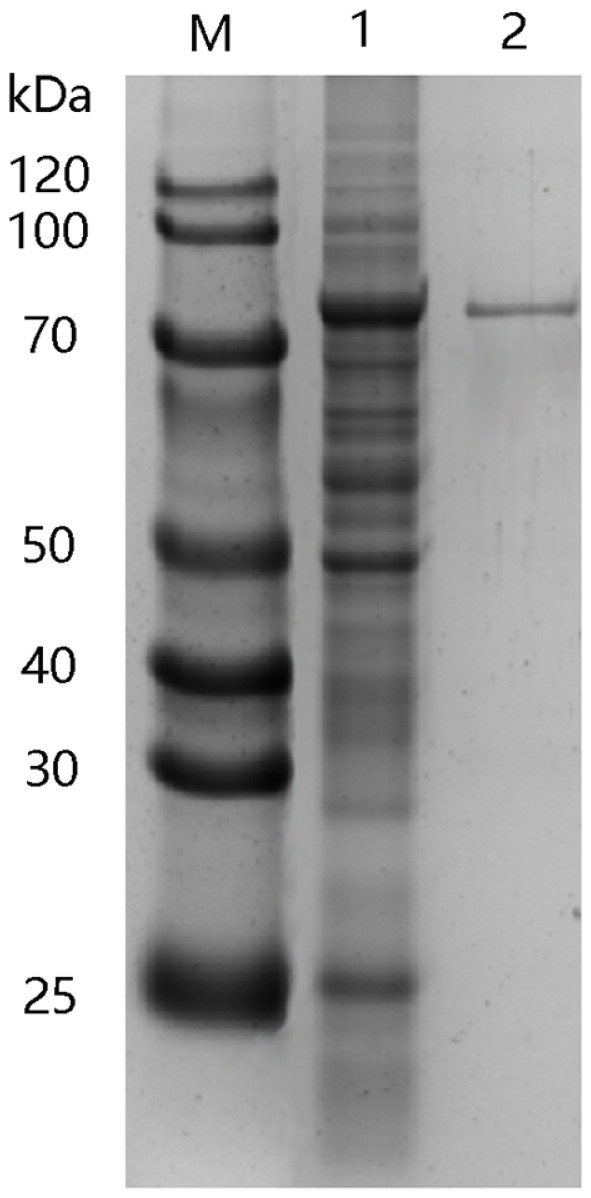
SDS-PAGE analysis of RsUGT expressed in *E. coli* BL21 (DE3). Lane M: protein molecular mass marker; lane 1: the soluble protein of *E. coli* BL21 (DE3) harboring pGEX-RsUGT at 30°C; lane 2: RsUGT purified by GSTSep Glutathione Agarose Resin GST affinity.

RsUGT was purified using a GSTSep Glutathione Agarose Resin GST affinity column, achieving a yield of 58.6%. The specific enzyme activity of the purified RsUGT was significantly enhanced, being 20.8 times higher than that of the crude enzyme, and reached 79.2 mU/mg (as shown in Table 1). SDS-PAGE analysis of the purified RsUGT confirmed its molecular mass to be approximately 77.7 kDa (Fig. 2, Lane 2).

**Fig. 3:**
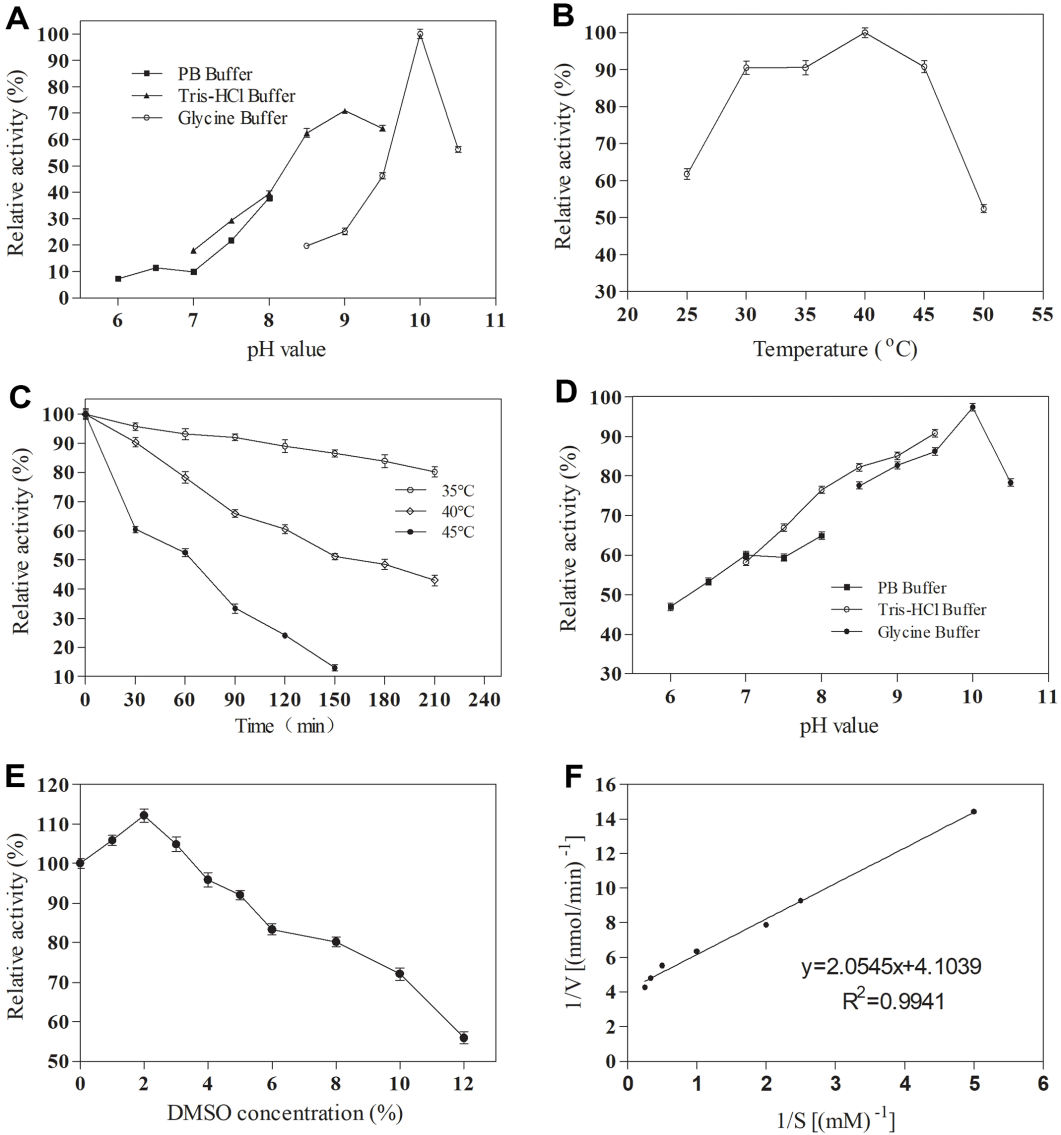
Characterization of purified enzyme RsUGT. (**A**) the effect of different pH on the activity of the purified enzyme RsUGT; (**B**) the effect of different temperature on the activity of the purified enzyme RsUGT; (**C**) the thermal stability of the purified enzyme RsUGT at 35, 40, and 45°C; (**D**) the pH stability of the purified enzyme RsUGT; (**E**) the effect of DMSO on the activity of the purified enzyme RsUGT; (**F**) Lineweaver-Burk plot of RsUGT activity with *p*HBA concentration.

In any enzyme-catalyzed reaction, pH and temperature are pivotal factors. Hence, the impact of pH and temperature on the purified RsUGT activity was investigated using *p*HBA as the substrate. As depicted in Fig. 3A, the optimum pH of RsUGT measured in glycine buffer and Tris-HCl buffer was 10.0 and 9.0, respectively. This phenomenon was also noted in other glycosyltransferase research reports [22]. The results showed that the enzyme was an alkaline enzyme. Regarding temperature, the highest relative enzyme activity of RsUGT was observed at 40°C (Fig. 3B), and the enzyme retained over 50% of its maximum activity within the temperature range of 25-50°C, which indicates that the enzyme has a wide temperature application range.

Given the importance of thermal and pH stability in industrial applications, the thermal stability of RsUGT was assessed at 35°C, 40°C, and 45°C. The results demonstrated that RsUGT can still maintain more than 80% residual enzyme activity at 35°C for 210 min (Fig. 3C), showing excellent thermal stability at 35°C. It also had good thermal stability at 40°C, and the half-life was about 150 min (Fig. 3C). However, the thermal stability was poor at 45°C, and the half-life was only 60 min. Furthermore, the pH stability of RsUGT was studied, and the results showed that the enzyme maintained good stability in buffers with a pH range of 6.5-10.5, with relative enzyme activity remaining above 50% (Fig. 3D).

Common metal ions and chemical reagents were chosen to assess their impact on RsUGT activity, with their concentrations in the reaction system meticulously maintained at 1 mM. According to Table 2, Cu^2+^, Hg^2+^, and Co^2+^ were found to completely inhibit the enzyme activity of RsUGT. Similarly, glycosyltransferase from *Gentiana triflora* has the same result [23]. Furthermore, Zn^2+^, Fe^2+^, and Mn^2+^ notably decreased the activity of RsUGT, while other metal ions also exhibited inhibitory effects. In contrast, ethylene diaminetetraacetic acid (EDTA), a metal chelating agent, had no significant impact on RsUGT activity, suggesting that free metal ions may not be crucial for maintaining its three-dimensional structure [24]. Additionally, dithiothreitol (DTT), a sulfhydryl inhibitor, significantly reduced RsUGT activity, hinting at the presence of sulfhydryl groups in the catalytic residue of RsUGT [25].

DMSO is known to be toxic to enzymes and can impact their activity. Therefore, the effect of various DMSO concentrations on RsUGT activity was investigated. As illustrated in Fig. 3E, within a DMSO concentration range of 1% to 2%, the activity of RsUGT increased slightly. At a DMSO concentration of 2%, the enzyme activity peaked, with an increase of 12.15%. However, as the DMSO concentration exceeded 3%, the enzyme activity decreased significantly, aligning with the findings reported by Pei *et al*. [23]. Consequently, a DMSO concentration of 2%was used in subsequent gastrodin biosynthesis.

Michaelis-Menten kinetics can elucidate the rate of an enzyme-catalyzed reaction based on the concentration of the enzyme and its substrate [26]. The values for kinetic constants of RsUGT were measured from the double reciprocal Lineweaver-Burk plot (Fig. 3F). The values of *K*_M_ and *V*_max_ were found to be 0.50 mM and 171.60 mU/mg, respectively. Consequently, the recombinant RsUGT had a good affinity for *p*HBA.

### Optimizing the Conditions for the Enzymatic Biosynthesis of Gastrodin

**Fig. 4:**
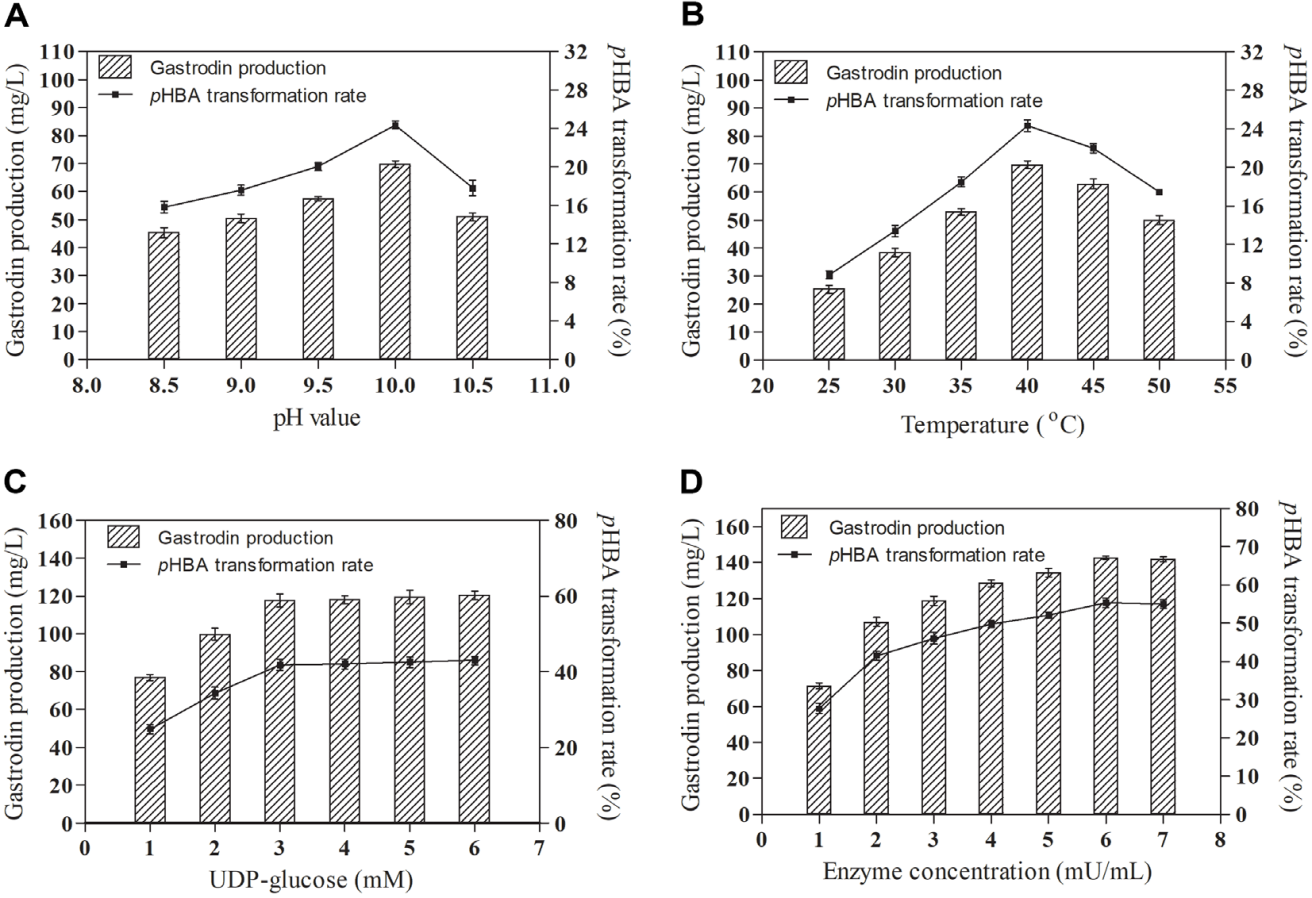
Optimizing the conditions for the enzymatic biosynthesis of gastrodin by RsUGT. Effects of pH(**A**), temperature (**B**), UDP-glucose concentration (**C**), and RsUGT concentration (**D**) on the gastrodin production and *p*HBA transformation rate.

Considering the significant effects of pH and temperature on enzyme activity, their effects on gastrodin biosynthesis were studied. As depicted in Fig. 4A, the highest gastrodin production and *p*HBA transformation rate were achieved at pH 10.0, with values of 69.70 mg/l and 24.35% respectively. This was attributed to the optimal pH of RsUGT being 10.0, where it exhibited excellent stability. Furthermore, as shown in Fig. 4B, the highest gastrodin production and *p*HBA conversion rate occurred at 40°C, reaching 69.71 mg/l and 24.35% respectively. UDP-glucose serves as a crucial sugar donor in the biosynthesis of gastrodin. Consequently, the effects of various UDP-glucose concentrations on gastrodin biosynthesis were investigated. Within a UDP-glucose concentration range of 1.0-3.0 mM, gastrodin production and *p*HBA conversion rate significantly improved, reaching 119.66 mg/l and 41.80% respectively (Fig. 4C). However, beyond a UDP-glucose concentration of 3.0 mM, the increase in gastrodin production and *p*HBA conversion rate slowed down (Fig. 4C). Therefore, the most suitable UDP-glucose concentration was determined to be 3.0 mM. Additionally, the effects of different enzyme concentrations on gastrodin production and *p*HBA conversion rate were also studied. As illustrated in Fig. 4D, gastrodin production and *p*HBA conversion rate increased with the increase in enzyme concentration, with the optimal enzyme concentration being 6 mU/ml. At this enzyme concentration, the gastrodin production and *p*HBA conversion rate reached 136.85 mg/l and 55.34% respectively.

### The Time Courses of Gastrodin Production

**Fig. 5:**
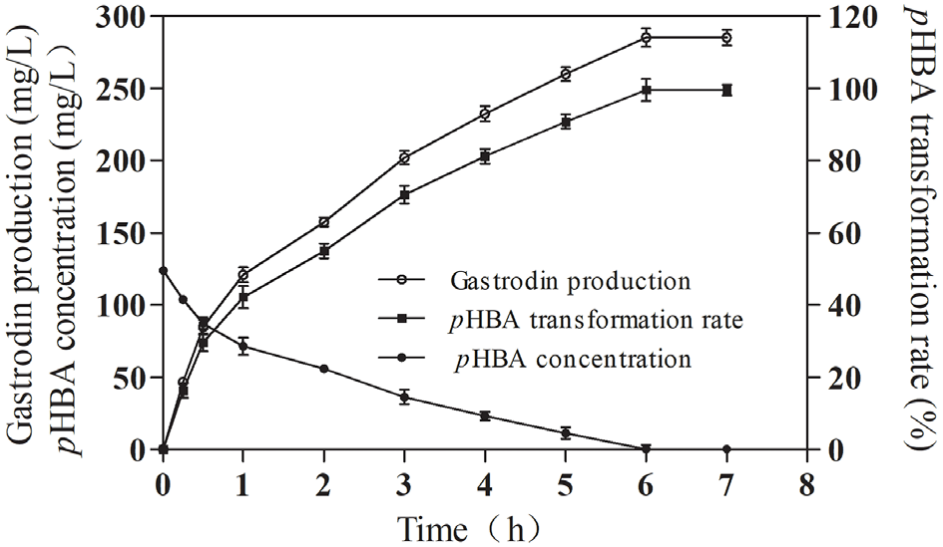
Time courses of gastrodin production, *p*HBA transformation rate, and *p*HBA concentration in an enzyme-catalyzed reaction system.

Using the optimum conditions of gastrodin biosynthesis established above, we investigated the changes in gastrodin production, *p*HBA conversion rate, and *p*HBA concentration over time. Within the first 0.25 h of biosynthesis, the specific productivity was observed to be 121.46 mg/l/h (Fig. 5). As the reaction progressed, the specific productivity gradually increased, peaking at 180.31 mg/l/h between 0.25 and 0.5 h (Fig. 5). However, beyond 0.5 h, the specific productivity began to decline, reaching 91.30 mg/l/h between 0.5 and 1 h. After 1 h of reaction, the specific productivity dropped rapidly, achieving 40.57 mg/l/h between 1 and 3 h and 27.70 mg/l/h between 3 and 6 h. At the end of 6 h, the highest gastrodin production was recorded at 285.35 mg/l, with a molar conversion rate of *p*HBA reaching 99.67%, which is the highest gastrodin production *in vitro* enzymatic biosynthesis so far. Previous studies Xia *et al*. on the *in vitro* biosynthesis of gastrodin using AtUGT from *Arabidopsis thaliana* reported a maximum production of 2.67 g/l [17], which is currently the highest reported *in vitro* biosynthesis of gastrodin. However, their reaction required 2 mg of AtUGT enzyme protein, resulting in a ratio of gastrodin production to protein input of 2.67 mg/mg. In contrast, the biosynthesis of gastrodin by RsUGT can reach a ratio of 3.74 mg/mg, which was 1.40 times higher than that of AtUGT. Therefore, compared with AtUGT, RsUGT demonstrated significant advantages in the *in vitro* biosynthesis of gastrodin.

### Optimization of the Conditions of Gastrodin Production by BL-RsUGT

**Fig. 6:**
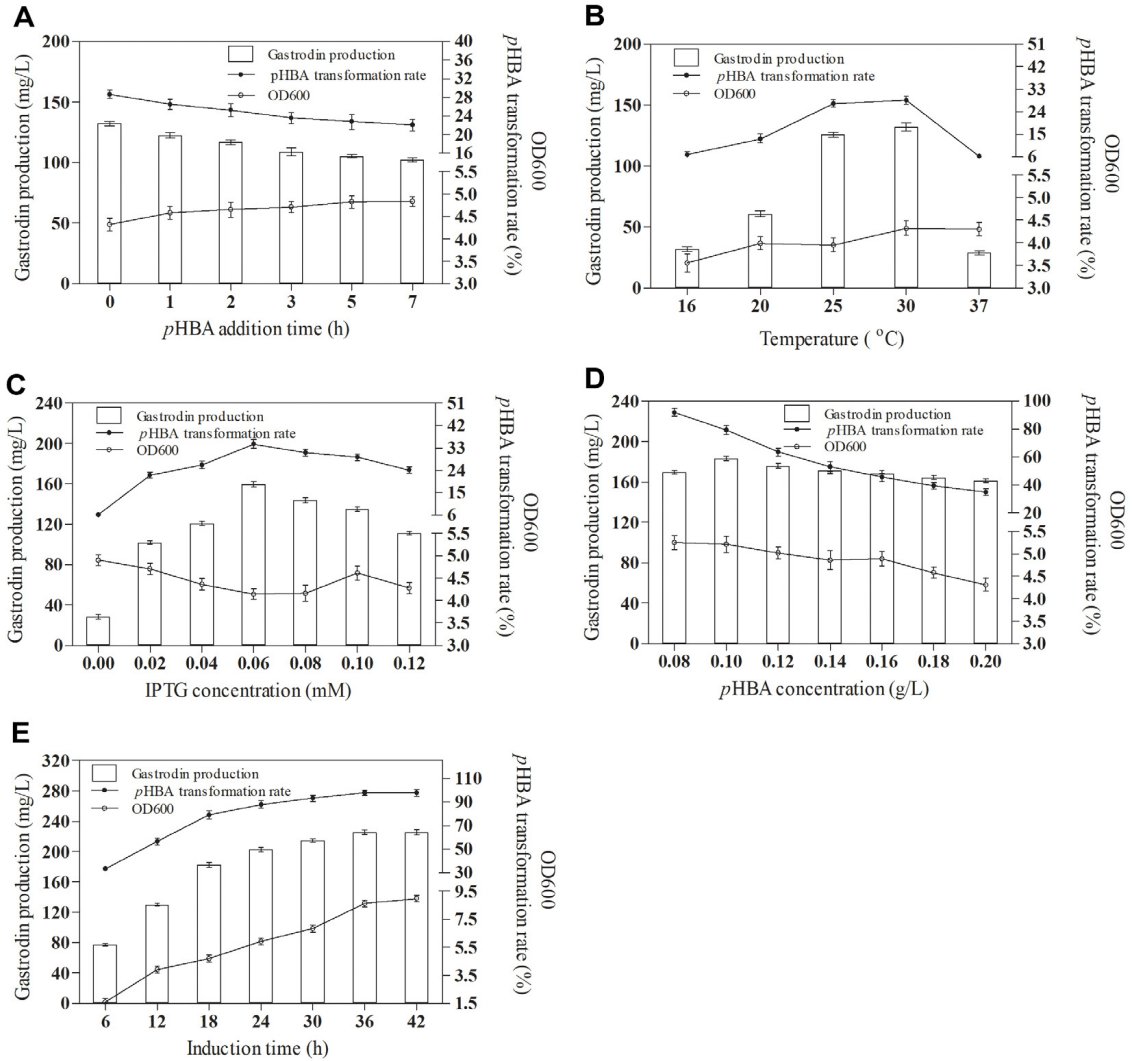
Optimization of the conditions of gastrodin production by BL-RsUGT. Effects of *p*HBA addition time (**A**) temperature (**B**) IPTG concentration (**C**) *p*HBA concentration (**D**), and induction time (**E**) on the gastrodin production and *p*HBA transformation rate.

Compared with enzyme catalysis *in vitro*, intracellular UDP-glucose can be used for gastrodin biosynthesis *in vivo*, thus eliminating the need to add expensive UDP-glucose. Therefore, it was of great research value to study the biosynthesis of gastrodin by recombinant strain BL-RsUGT. To optimize the biosynthesis of gastrodin by recombinant strain BL-RsUGT *in vivo*, the effects of various *p*HBA addition times on gastrodin production were studied. As shown in Fig. 6A, the optimal *p*HBA addition time of recombinant strain BL-RsUGT was 0 h, in other words, it was added to the culture solution together with inducer IPTG. The corresponding gastrodin production and *p*HBA transformation rate were 131.21 mg/l and 28.45% respectively. Moreover, the effects of various induction temperatures on gastrodin production were studied. As shown in Fig. 6B, the optimal induction temperature of recombinant strain BL-RsUGT was 30°C, and the corresponding gastrodin production and *p*HBA transformation rate were 132.20 mg/l and 28.66% respectively, which were much higher than the results at 37°C. This was because higher temperature would cause incorrect folding of protein, and then affected the biosynthesis of gastrodin. The expression of intracellular protein of recombinant strain BL-RsUGT needs inducer IPTG, but high concentration of IPTG can lead to incorrect protein folding, and finally form inactive inclusion bodies [27]. Therefore, the effects of various IPTG concentration on gastrodin production were studied. As shown in Fig. 6C, the optimal IPTG concentration induced by recombinant strain BL-RsUGT was 0.06 mM, and the corresponding gastrodin production and *p*HBA conversion rate were 159.67 mg/l and 34.62% respectively. Furthermore, the effects of various *p*HBA concentrations on the gastrodin production were also studied. As shown in Fig. 6D, with the increase of *p*HBA concentration, the gastrodin production first increased and then decreased. When the concentration of *p*HBA was 0.1 g/l, the highest gastrodin production was 183.14 mg/l, and the corresponding conversion rate of *p*HBA reached 79.41%. According to the change of the corresponding OD_600_ value, it can be seen that higher concentration of substrate *p*HBA was unfavorable to the growth of *E. coli*, which was consistent with the research results of Cui *et al*. [16]. In addition, it may also be related to the substrate uptake level or UDP-glucose cell concentration limit. Subsequently, the changes of gastrodin production and *p*HBA transformation rate with time were also studied. As shown in Fig. 6E, with the passage of time, the gastrodin production and *p*HBA transformation rate increased continuously. When the induction time was 36 h, the gastrodin production reached the maximum, with the highest production of 225.99 mg/l, and the corresponding *p*HBA transformation rate reached 98.00%, which was higher than the results studied by Cui *et al*. [16].

## Conclusion

In this study, an alkaline UDP-glycosyltransferase from *Rauvolfia serpentina* (RsUGT) was expressed in *E. coli* BL21(DE3). The optimum temperature was 40°C, and the optimum pH of RsUGT was 10.0. The values of *K*_M_ and *V*_max_ were found to be 0.50 mM and 171.60 mU/mg respectively, and 2% DMSO could increase the RsUGT activity by 12.15%. Under the optimal enzymatic catalysis conditions, the highest gastrodin production was 259.54 mg/l, accompanied by a molar conversion rate of 99.7%. In addition, under the optimal biosynthesis conditions of recombinant strain BL-RsUGT, the highest gastrodin production was 225.99 mg/l, and the corresponding *p*HBA conversion rate was 98.00%. The results showed that RsUGT has potential application value in the preparation of gastrodin.

## Figures and Tables

**Table 1:** Purification process for the recombinant protein RsUGT.

Purification step	Total protein (mg)	Total activity (mU)	Specific activity (mU/mg)	Yield (%)	Purification factor (fold)
Crude extract	231.2	878.5	3.8	100.0	1.0
GSTSep Glutathione Agarose Resin GST affinity	6.5	514.8	79.2	58.6	20.8

**Table 2:** Effects of metal cations and reagents on RsUGT Activity.

Cation and reagent^a^	Relative activity (Mean% ± SD)	Cation and reagent^a^	Relative activity (Mean% ± SD)^a^
Control	100.0 ± 0.2	Ca^2+^	97.0 ± 0.2
Li^+^	76.2 ± 0.4	Zn^2+^	33.1 ± 0.3
K^+^	74.9 ± 0.3	Ba^2+^	97.0 ± 0.3
Na^+^	89.2 ± 0.4	Fe^2+^	28.3 ± 0.1
NH_4_^+^	78.1 ± 0.3	Hg^2+^	0 ± 0
Mn^2+^	20.7 ± 0.1	Fe^3+^	96.0 ± 0.4
Mg^2+^	85.3 ± 0.6	Al^3+^	97.0 ± 0.5
Cu^2+^	0 ± 0	EDTA	97.2 ± 0.3
Co^2+^	0 ± 0	DTT	51.9 ± 0.2

Values shown are the mean of duplicate experiments, and the SD represents the standard deviation.

^a^Final concentration of substrate was 1.0 mM.
